# Medication reconciliation at hospital admission and discharge: insufficient knowledge, unclear task reallocation and lack of collaboration as major barriers to medication safety

**DOI:** 10.1186/1472-6963-12-170

**Published:** 2012-06-21

**Authors:** Nelleke van Sluisveld, Marieke Zegers, Stephanie Natsch, Hub Wollersheim

**Affiliations:** 1 Scientific Institute for Quality of Healthcare (IQ healthcare), Radboud University Nijmegen Medical Centre, PO Box 9101, Nijmegen, the Netherlands; 2 Department of Clinical Pharmacy, Radboud University Nijmegen Medical Centre, PO Box 9101, Nijmegen, the Netherlands; 3 Scientific Centre Quality of Care, Catholic University Leuven, Kapucijnenvoer, B-3000, Leuven, Belgium

**Keywords:** Adverse events, Safety, Quality, Medication reconciliation, Medication error, Implementation, Implementation barriers

## Abstract

**Background:**

Medication errors are a leading cause of patient harm. Many of these errors result from an incomplete overview of medication either at a patient’s referral to or at discharge from the hospital. One solution is medication reconciliation, a formal process in which health care professionals partner with patients to ensure an accurate and complete transfer of medication information at interfaces of care. In 2007, the Dutch government compelled hospitals to implement a bundle concerning medication reconciliation at hospital admission and discharge. But to date many hospitals have failed to implement this bundle fully. The aim of this study was to gain insight into the barriers and drivers of the implementation process.

**Methods:**

We performed face to face, semi-structured interviews with twenty health care professionals and managers from several departments at a 953 bed university hospital in the Netherlands and also from the surrounding community health services. The interviews were analysed using a combined theoretical framework of Grol and Cabana to classify the drivers and barriers identified.

**Results:**

There is lack of awareness and insufficient knowledge of health care professionals about the health care problem and the bundle medication reconciliation. These result in a lack of support for implementing the bundle. In addition clinicians are reluctant to reallocate tasks to nurses or pharmacy technicians. Another major barrier is a lack of communication, understanding and collaboration between hospital and community caregivers. The introduction of more competitive market forces has made matters worse. Major drivers are a good implementation plan, patient awareness, and obligation by the government.

**Conclusions:**

We identified a wide range of barriers and drivers which health care professionals believe influence the implementation of medication reconciliation. This reflects the complexity of implementation. Implementation can be improved if these factors are adequately addressed. The feasibility and effectiveness of these strategies should be tested in controlled trails.

## Background

Medication errors are one of the leading causes of patient harm in hospitals. In over half of the patients discrepancies were found between the medication patients were taking at home and the list of medications known to the hospital caregivers after intake [1-4]. These discrepancies are caused mostly by incomplete medication history taking at admission or by an incomplete handover of medication information between the community and hospital caregivers. This results in an incomplete overview of medication and an interrupted, or incorrect, drug treatment [5,6]. This subsequently may result in adverse drug events for patients, which could ultimately lead to life-threatening situations, avoidable treatments, re-admissions to hospital, and substantial costs [7-12].

Medication reconciliation is the formal process in which health care professionals partner with patients to ensure an accurate and complete transfer of medication information at interfaces of care. It is an internationally accepted strategy to reduce medication errors at patient transfers [1,12]. Medication reconciliation at admission involves a systematic process in order to obtain a complete and accurate list of a patient’s current home medications. These include all prescription medications and over-the-counter drugs as well as herbals, vitamins, supplements, vaccines, parenteral nutrition, and blood derivatives. Medication information is gathered from different sources: the patient, his or her relatives, the medical hospital record, the patient’s community pharmacy, the general practitioner (GP), and other community caregivers [12-14]. Medication reconciliation at hospital discharge means that newly prescribed, continued, discontinued and modified medications as well as the reasons for those changes are communicated to pharmacists and other community caregivers. Moreover, patient counselling is used to inform the patient about his or her old and new medications, about any reasons for changing its duration, frequency, route, and dose, and about the time the medications should be taken [13,14].

Through medication reconciliation errors of inadvertent omission of medications needed at home, failure to restart home medication after discharge, duplication of therapy at discharge, errors associated with incorrect doses or timing, and adverse drug-drug or drug-disease interactions can be avoided [1,15]. Medication reconciliation intercepts a significant number of discrepancies. It decreases the rate of medication errors, reduces potential adverse drug events, and thus reduces work and re-work [16-19]. Medication reconciliation is an important theme in several national patient safety campaigns [12,20-23].

While the process seems straightforward, implementing medication reconciliation at hospital admission and discharge has proven to be very difficult. Studies in the U.S. showed that the medication reconciliation process of gathering, organising, and communicating medication information is complicated by several factors. Not least are the number of disciplines involved in the process: clinicians, nurses, hospital pharmacists, community pharmacists, community caregivers and patients themselves [24-27]. Vague agreements or no agreements at all about the tasks of every person involved lead to inefficiencies and a failure to implement sufficiently [28].

A systematic insight into the factors that influence the implementation process is lacking. Therefore this study aims to gain insight into the barriers and drivers of this process. It adopts the perspective of the health care professionals and uses a theoretical framework derived from the field of implementation science. Comparable articles, all from the U.S., focus solely on organisational aspects and barriers to the patient. By adopting the theoretical framework, we will research a broader spectrum of factors influencing implementation, for example characteristics of the innovation, attitude of health care professionals and the economic, legal and political context. This insight could be used to optimise the medication reconciliation process at hospital admission and discharge with implementation strategies tailored to the barriers and drivers found.

## Methods

### Setting

The study was performed in a 953 bed university hospital and the surrounding community. The Dutch health care system is mainly based on a competitive regulated market. The allocation of care and the price of individual treatments are determined by the market. The government uses a regulatory framework to achieve affordable health insurance and good quality of health care. Patient safety is a high priority for the government, hospitals and other health care professionals in the Netherlands.

### The Dutch bundle intervention for medication reconciliation

In 2007, a Patient Safety Programme was launched in Dutch hospitals, which included a bundle intervention concerning medication reconciliation at hospital admission and discharge [23]. In the following paragraph this bundle has been summarised. 

Medication reconciliation on admission

Collect information on the medication history from the community pharmacy

Interview the patient by a trained professionala about medication use and history

Create an up-to-date and complete list of the patient’s current medications

Medication reconciliation on discharge

Create an up-to-date medication list based on data from the hospital pharmacy, and the hospital’s medical record

Write the discharge prescription medication list authorised by the clinician responsible

Undertake patient counselling by a trained professional (a pharmacist, pharmacist assistant, nurse, pharmaceutical consultant or a pharmacy practitioner) at discharge

Ensure handover of an up-do-date medication list, discharge prescription, as well as information about medication which were discontinued and changed and the reason for this, to the community pharmacy, general practitioner and other health care organizations

The bundle has been developed by an expert group, including several types of medical specialists, nurses, pharmacists, and policy makers. In addition, several professional associations were involved, among others the association of general internists, nurses, cardiologists, paediatricians, hospitals, hospital pharmacists, and geriatrics. It was based on available international literature, guidelines, safety campaigns, and best clinical practices. Since 2011, medication reconciliation at hospital admission and discharge has been made compulsory by the government for every planned hospital admission and discharge. The implementation of the bundle is monitored by the Dutch Health Care Inspectorate using indicators, which are measured by the hospital themselves. Medication reconciliation is one of ten clinical innovations to be implemented within the Dutch Patient Safety management programme for hospitals.

The board of the hospital in this study assigned one professional (SN) to facilitate the implementation of medication reconciliation. The hospital pharmacy developed the protocol and forms. Individual departments, however, are responsible for implementing medication reconciliation themselves.

### Study design

A qualitative research perspective was used for both a wide and a detailed exploration of the barriers and drivers to the implementation of medication reconciliation. We conducted face to face interviews from December 2010 to May 2011. We aimed, in particular, to investigate factors which influence the implementation process according to the individual perceptions of the persons involved. Formal ethical approval was, according to the Dutch law, not needed for this study.

### Interview participants

To ensure maximum variation in participants and their perceived barriers and drivers the principles of ‘purposeful sampling’ were applied [29]. In order to achieve a wide exploration of all factors influencing this implementation, we invited physicians, nurses, and hospital and community pharmacists who were involved in the implementation of medication reconciliation in their daily routine [30]. In addition we included a policy maker who advises health care professionals on quality and safety issues and a quality researcher who observed, on several wards, how physician and nurses carry out the different steps of the medication reconciliation process. Clinicians and nurses were invited from seven departments that were in the process of implementing medication reconciliation to a greater or lesser extent, including internal medicine, surgery, paediatrics, pulmonary diseases, orthopaedics, neurology, and cardiology. The number of interviews depended on reaching saturation that is when no new barriers or drivers had been identified.

### Data collection

The interviewees were informed about the study and its aim by email. At the beginning of the interview, the interviewees confirmed their willingness to participate and gave verbal informed consent. The interviews lasted around 50 minutes.

The interviews were semi-structured, containing open questions about specific themes based on the theoretical framework (see data analysis). This enabled the interviewees to talk freely, allowing them to elaborate their personal feelings about the barriers and drivers they experienced. After some introductory questions about the bundle and its implementation, three main questions were asked: ‘According to your experiences, which factors bar the implementation of medication reconciliation at hospital admission and discharge?’, ‘Which factors drive the implementation?’ and ‘How could the implementation be improved?’ Asking open-ended questions allowed the interviewees the freedom to elaborate on those factors that were perceived as most important. Subsequent questions were then asked in order to discuss the factors in more depth and to explore other factors from the theoretical framework (see data analysis).

### Data analysis

The interviews were audio taped and transcribed verbatim. A thematic analysis was performed. The data were grouped into previously formulated themes and subthemes of a combined theoretical framework for barriers and drivers to implementation (Table 1). The framework was based on ‘the implementation model’ of Grol and ‘the framework for improvement’ of Cabana for the classification of the barriers and drivers identified [31-33]. According to this framework, the implementation of medication reconciliation can be hindered or facilitated by factors related to the innovation, health care professionals, patients, the organisation, and to the social, political, legal, and economic context.

**Table 1:** **Theoretical framework for classifying barriers and drivers, based on Grol and Cabana**[26-28]

**Levels**	**Sublevels**
Innovation	Complexity, Compatibility, Credibility, Accessibility, Amount of information, Feasibility, Attractiveness, Advantage, Utility, Usefulness
Health care professionals	Cognition, Awareness, Attitude, Motivation to change, Knowledge, Education
Patients	Compliance, Polypharmacy, Multiple co-morbidity, Knowledge, Skills, Attitude
Social context	Culture of social network, Opinion of colleagues, Leadership, Collaboration, Social learning
Organisation	Organisation of care processes, Organisational structure, Time, Staff, Capacities, Resources, ICT infrastructure
Economic context	Financial support
Political and legal context	Social developments, Political developments and policies, Legal obligations and regulations

Two researchers (NvS and MZ) analysed the transcripts independently using the framework. If a barrier or driver identified could not be placed within an existing subtheme, then a new subtheme was formulated. The discrepancies in classification between the two researchers were discussed until a consensus was reached. The software programme Atlas.ti 6.0 was used to facilitate the classifying process.

## Results

### Description of participants

Twenty participants were invited for an interview: four clinicians, ten nurses, two hospital pharmacists, two community pharmacists, one policy maker, and one quality researcher. Sixty per cent was female. Of the 14 clinicians and nurses, four participants worked at the department of paediatrics, three in internal medicine, two in surgery, two in cardiology, one in pulmonary diseases, one in orthopaedics, and one in neurology. The results of the interviews are summarised in Table 2. Below, the most prominent quotes from the interviewees and a summary of the findings of all the interviews are given.

**Table 2:** Perceived barriers and drivers to the implementation of medication reconciliation

**Levels**	**Perceived Barriers**	**Perceived Drivers**
**Innovation**		
Usefulness	The bundle does not meet the wishes or needs of professionals	Bundle creates more clarity about medication
Complexity	Complex process, many professionals involved	Clear written manual and protocol of bundle
Compatibility		Tailoring bundle to individual departments or specialities
Credibility	Lack of evidence of the effectiveness of the bundle	
**Professionals**		
Knowledge	Insufficient knowledge of the health care problem, the bundle,	
	benefits of innovation, best performance and generating feedback	
	Not convinced that innovation leads to better and more efficient care	
Cognition	Do not recognize the care problem	
	Physicians prefer to conduct medication reconciliation themselves	
Awareness	Resistance to the imposed way of working	Creating awareness of the health care problem by process mapping
Attitude	Shifting responsibilities	Quality and safety are seen as important
		Involve all professionals, including community caregivers
**Patients**		
Knowledge	Limited knowledge of their medications	Encourage patient empowerment through education
Awareness		Increase the awareness and responsibility for, carrying an up-to-date medication list
Attitude	Patient has other needs or priorities	
**Social context**		
Social learning	Top down implementation results in less involvement of departments and professionals	Snowball effect of best practice
Collaboration	No collaboration or arrangements between departments and	Having a multidisciplinary project group in charge of the
	hospital and community caregivers	implementation
	Information from community pharmacies is not available during out of office hours	Regional collaboration and agreements
Leadership	No sanction for departments who do not implement the bundle	The reinforcement and support of the bundle by management
		Good and clear leadership
Competition		Competitive spirit between departments
**Organisation**		
Implementation resources	Extra resources not being available for adhering to the bundle and to measure indicators	Adopting a phased approach to implementation
		Investing time, effort and resources
		Having a detailed implementation plan
		Clear and uniform forms and protocols
Chain of care	Medication reconciliation not being implemented at every transfer or in related departments	
Task reallocation	No agreements regarding tasks and responsibilities	Clear descriptions of roles, tasks and responsibilities
		Task reallocation to and more involvement of pharmacy technicians
Staff	High turnover of personnel and interns	Protocol for new personnel
Feedback	Quality indicators are not measured, no feedback information available	Create an evaluation and feedback mechanism
		A central incident reporting system for both hospital and community caregivers
Feasibility	Simultaneous implementation of multiple safety interventions	
ICT		Digital support for implementation, measurement and feedback of quality indicators
		Regional or national electronic medication patient file
**Economic, political and legal context**		
Economic	Market forces result in competition for tasks and funding among care professionals	
Political	Social pressure to save money	Patient safety is an important political subject
Legal	Uncertainty about patient privacy	Obligation by government
	Undersigning the discharge medication list implies a legal	Reinforcement by the Health Care Inspectorate
	responsibility for all prescribed medication	

### Perceived barriers to the implementation of medication reconciliation

#### Innovation

"“We do not have rock hard evidence that this bundle will for example prevent death in a number of patients. It is more like a common sense measure.” – policy maker -"

The motivation of professionals was influenced by the lack of evidence from randomised controlled trials of the effectiveness of the bundle medication reconciliation. Due to the rather thin evidence it was not possible for policy makers to impose one specific method of medication reconciliation. This caused uncertainty.

"“My experience with this bundle is that it is pretty free. It provides a direction, but the rest should be filled in by the professionals themselves.” – nurse -"

The professionals stated that the bundle left a gap between the recommended care and how this level of care should be reached.

"“Departments are not aware that they have to rearrange their way of working to make this change permanent.” – policy maker -"

Performing medication reconciliation is a complex and comprehensive task. Proper implementation requires both investment of resources and reorganisation of current care processes if its integration is going to be sustained in routine practice.

#### Health care professionals

"“I think there was an investigation by the hospital pharmacy about medication errors. It showed that our department performed really well. So I think there was not much need to change.” – nurse -"

Some professionals were not convinced that medication reconciliation resulted in better care within their department. They did not recognise the care problem.

"“We, nurses, do history taking in which we also ask patients about their medications. The physician also asks patients about their medications. A clinician does not blindly accept the information of an ‘educated professional’. In all cases, the clinician makes sure the medication reconciliation is correct. So medications are discussed and noted twice.” – nurse -"

"“The clinician is ultimately responsible, but a nurse could also perform this interview. The responsibility, qualification and competence to perform such a task does not need to be done by one professional only.” – hospital pharmacist -"

Clinicians do not want to reallocate certain tasks to other professionals. They prefer to carry out medication reconciliation themselves, because they believe that it should be the task of clinicians and because they are ultimately responsible for their patients’ medications. Even if it has been decided on departmental level that a nurse or a pharmacy technician should perform the medication history taking, physicians would still ask the patient about his or her medication, because they do not entirely trust the results of others.

#### Patients

"“Often, the patient does not want to wait for the counselling at discharge, he just wants to go home.” – clinician -"

Numerous patients have limited knowledge about their medication, which makes medication history taking more complicated. Moreover, most patients want to go home as soon as possible and therefore they give possibly less priority to being educated about their medications.

#### Social context

"”Departments all have their own way of working. We have to see how medication reconciliation can fit in. This leads to an obstacle, because if you let departments choose for themselves, each department will choose differently. Alignment should be improved, and the whole process should be standardised.” – policy maker -"

No, or unclear, agreements and a lack of collaboration about tasks and responsibilities exist between departments, between regional hospitals, and between hospital and community caregivers and especially, community pharmacists. An example of bad alignment is the fact that information from community pharmacists is not available for hospitals during evening hours and weekends.

"“Departments have to report the progress towards implementation in quarterly meetings with the board. The hospital board does not, however, sanction departments.” – policy maker -"

There are no sanctions for departments who are not actually implementing the bundle. Professionals and departments do not receive feedback on bad performance and there are no sanctions to encourage professionals to improve bad performance.

#### Organisation

"“We are changing existing structures, because we want physicians to act differently. This is fairly intensive. Furthermore, we cannot expect departments to implement ten safety themes at once.” – policy maker -"

Professionals report that they were overwhelmed with following care innovations which follow rapidly, one after the other. The Dutch Patient Safety Programme consists of ten themes, of which medication reconciliation is only one. There was no financial compensation for the time invested in carrying out the implementation, nor for the reorganisation of the care required or the measurement of quality of health care indicators to evaluate the implementation process.

"“Error reports are mostly not about the discharge process, because we do not know what happens to the patient afterwards.” – nurse -"

Feedback about patient harm as a result of poor medication reconciliation at discharge was not provided to hospital caregivers, as they lose sight of the patients once they have been discharged.

"“The tasks and responsibilities are unclear regarding interviewing the patient about his or her medication.” – hospital pharmacist -"

The tasks, roles and responsibilities are not clearly defined among professionals. This leads to inefficiencies, because the same tasks are being performed twice or even more.

"“It is important that medication reconciliation starts in the outpatient clinic. They should give the current medication list to the anaesthesiologist, and the anaesthesiologist should give the information to the department where the patient will be treated. Then, they will check if the medication list is correct. This is how it should happen.” – nurse -"

There are no agreements about when medication reconciliation should take place and the bundle is not yet implemented at every hospital department. If medication reconciliation is not performed at every transfer, the medication list at the point of discharge will be inaccurate.

#### Economic, political and legal context

"“I am responsible for all medications, including those prescribed by other clinicians, simply because of this one signature on the discharge medication form.” – clinician -"

Uncertainties about legal accountability as well as privacy matters made health care professionals feel insecure.

"“We are obliged to have each patient’s permission. We will be reprimanded by the Health Care Inspectorate if we do not receive this permission.” – community pharmacist -"

"“The opinion of some pharmacists about privacy is very overrated. Medication safety is more important. Up to now, if I ask a patient, they all agree to sending the medication list (to the hospital).” – community pharmacist -"

Some community pharmacists did not send the medication list to hospital caregivers if they did not have explicit permission documented by the patient’s signature. Others did not weigh privacy as high as the medication safety of the patient, and would therefore send the medication list to the hospital in case of emergency, with or without the signature of the patient.

"“Community pharmacists may regard the hospital performing medication reconciliation as if they want to take tasks away from them. This could be an obstacle for optimal contact between the hospital and the community pharmacies. Pharmacies have been financially stripped in the last 2–3 years, and some are even making losses. The medication review means income. Hospitals should allow these people to make a living, because their work is important.” – community pharmacist -"

An economic factor which influences the relationship between, and collaboration with, community and hospital pharmacists is the financial compensation for carrying out medication reconciliation. Pharmacists and insurance companies are debating whether it should be covered through insurance.

### Perceived drivers to the implementation of medication reconciliation

#### Health care professionals

"“If you organise it in a proper way then the patient receives the correct medication, there are no errors made and it is less work.” – nurse -"

Involvement of professionals with both a proactive attitude and an awareness of the importance of medication reconciliation will support the implementation of the bundle.

"“Start the implementation by mapping out the process. This gives professionals insight into their performance; when are professionals performing medication reconciliation, which professionals perform it; and how much time is spent on it. This knowledge clarifies where to improve efficiency.” – hospital pharmacist -"

Process mapping will improve the awareness of professionals about the health care problem and will show the need for improvement to avoid inefficiencies in their daily practice. This will motivate professionals to adapt their routine to the bundle.

#### Patients

"“For two years, we (community pharmacists) have been alerting patients to take a medication overview when they have to go to a hospital.” – community pharmacist -"

Some community pharmacists improve patients’ awareness of medication safety as well as the patients’ responsibility about their own care. They provide high risk patients with an up-to-date medication list and emphasise the importance of always carrying an up-to-date medication list with them.

#### Social context

"“We were one of the worst performing departments, but we want to be top performers again.” – clinician -"

In the social context of a hospital, professionals have a competitive spirit. If a department performs medication reconciliation more effectively, other departments are likely to change their way of work to be just as, or more, effective.

"“People have to see that the innovation is supported by the leader. The hospital board showed leadership when they compelled all departments to implement the bundle.” – policy maker -"

Leadership is a driving factor. Leaders should be identifiable, present, approachable, enthusiastic, visible, and they should clearly endorse this care innovation. The reinforcement and support of the implementation of the bundle by the hospital board, head of departments and clinicians is important.

"“The content is the responsibility of the implementation content leader, but the responsibility of the process lies with the departments themselves.” – policy maker -"

The hospital board assigned one person to translate the intervention into protocols, forms and an implementation guide. This was intended to facilitate the whole implementation process. However, the responsibility for the implementation of the bundle was directed to the head of the department that is the physician in charge. The hospital board believes that decentralising the responsibility and approach will support the implementation.

#### Organisation

"“I think, based on the costs-quality ratio, that hospital pharmacy technicians are best suited to perform medication reconciliation, compared to clinicians, nurses and hospital pharmacists, and they should be the link between clinician, patient and pharmacy.” – community pharmacist -"

Task reallocation is an important driver. Interviewees indicate that hospital pharmacy technicians should play a larger role in the medication history taking.

"“A review should be done by the community pharmacy; a hospital pharmacy is not fit for such a task at all. The hospital pharmacy does not know the GP, and has no connections with him or her, which we do have. They cannot walk into the GP’s office, which we can and do.” – community pharmacist -"

Community pharmacists can play an important role in medication reconciliation, because they have greater and closer contact with the patient. Moreover, they have better insight into the comorbidities and medication history of the patient and are in closer contact with GPs and other community caregivers.

"“The implementation is an important phase. Making a good start is necessary for getting medication reconciliation embedded into the working process. We take on the implementation challenge with the whole department: nurses, physicians, etcetera, and discuss with each other how to implement it in this particular department.” – hospital pharmacist -"

An in-depth implementation plan, developed by a multidisciplinary team, is important and should include the following aspects according to the interviewees: an intervention tailored to local barriers; realistic objectives; clear leadership; and a clear start and end point of the project. Furthermore, a phased approach towards implementation was appreciated.

"“Reports about medication errors should be given as feedback to professionals, otherwise people will return to their former way of working. But if they see that fewer medication errors are made, that will certainly motivate them to continue doing medication reconciliation. Another thing that should be given as feedback is whether or not medication reconciliation is performed correctly, so that we can learn from it - a kind of self-learning system.” – clinician -"

Evaluation and feedback through indicators drives the improvement of the implementation.

"“In the evening, community pharmacies are closed. Insight into the electronic files of community pharmacists would help us enormously. Otherwise, clinicians have to prescribe without medication history.” – nurse -"

Several hospital and community caregivers said access to a reliable regional or nationwide electronic patient medication files for hospital and community caregivers would decrease the number of medication errors.

#### Economic, political and legal context

"“I know we will not escape from the implementation and we will just have to do it, because it is a legal regulation.” – clinician -"

An important political driver is the fact that patient safety is high on the political agenda. Therefore, the Dutch Patient Safety Programme, including medication reconciliation, has been made compulsory. The Health Care Inspectorate monitors if medication reconciliation is implemented in hospitals by using indicators.

## Discussion

Medication reconciliation is a method for reducing medication errors, patient harm and costs. In this study we showed that insufficient knowledge of care professionals, unclear task allocation, and a lack of collaboration within, and between, inpatient and outpatient settings are important barriers from the perspective of the health care professionals. On the other hand, health care professionals highlight drivers, such as a good implementation plan, patient empowerment, and obligation by the government, as benefiting the implementation. The barriers and drivers we identified can help to develop strategies for improving the implementation of medication reconciliation.

Our study found several barriers to the implementation of medication reconciliation. Firstly there was a lack of awareness as well as insufficient knowledge of health care professionals. Noticeable was the lack of awareness about the health care problem. Professionals do not know how many medication errors are made and the impact these can have on the wellbeing of the patient. Knowledge of the bundle and how best performance can be achieved was insufficient. Professionals did not recognise the positive impact that the bundle would have on their everyday care. It was not clear how quality indicators to evaluate the implementation of the bundle should be measured, registered or given as feedback to professionals.

Secondly the necessity of reallocation of tasks was not clear. Currently, there are no clear agreements about tasks and responsibilities, despite the fact that the bundle was released as early as January 2007. The bundle did not explicitly state who, where and when to perform the different parts of medication reconciliation. There were several opinions among professionals on how best performance should be reached within the process of medication reconciliation. Various studies conclude that medication history taking by pharmacists or pharmacy technicians results in fewer errors compared to history taking by clinicians [1,16]. Despite this, clinicians, in particular clinicians from non-surgery specialities, were opposed to reallocating their tasks. Their unwillingness originates from their autonomous way of working. Clinicians feel they should undertake this task, not least because ultimately as a clinician they are legally responsible for the complete treatment of the patient. Professionals indicate that when tasks are performed by someone other than the person responsible, it will result in uncertainties. Often clinicians do not trust the medication list if a nurse, pharmacy technician or pharmacists has done the history taking. All this resulted in inefficiencies and in different ways of working in the various departments. This complicated medication reconciliation at hospital admission, at transfers within the hospital and on discharge.

The third barrier is the impact market competition has on communication, understanding and collaboration. The relationship between community and hospital caregivers has become worse since the introduction of market-based competition in the Dutch health care system. Community pharmacies are more reserved in communicating medication information to other pharmacies. This is because, in their opinion, this information could also be used to lure patients with multiple medications to those competing pharmacies. Pharmacies gain most of their income from those patients. Since many hospitals currently also include hospital pharmacies, community pharmacies are equally reserved in sharing medication information with those hospitals. The probability exists that in the future performing medication reconciliation will be reimbursed by insurance. But this too would not encourage the cooperation between community and hospital pharmacists. They both want to do the job, because it is profitable. A lack of communication, understanding and collaboration between hospital caregivers and community caregivers is an important barrier to the medication reconciliation process [34,35].

An important driver found in our study was obligation by the government. It is obligatory to perform medication reconciliation in every Dutch hospital. The attitude of professionals changed when they had no choice but to implement it into their work. The hospital management reinforced the obligation of the government by assigning responsibility for the implementation to departmental heads and installing a professional who facilitates the process. There were indicators formulated, as described in the method section, to monitor the implementation of the bundle. Up to now, however, departments who do not co-operate have not been sanctioned.

Secondly, several interviewees mentioned the importance of a planned phase of implementation. A multidisciplinary team should be involved from the start comprising all stakeholders in the implementation of medication reconciliation. In particular this should include community care professionals such as community pharmacists. This team should standardise the process of medication reconciliation through the development of protocols and forms, which include all the wishes and needs of the professionals involved. If the implementation phase is carefully planned, the process of medication reconciliation standardised and the environment in which the intervention is implemented taken into account, then it is more likely to succeed.

Thirdly, patient awareness should be improved. Professionals indicate that medication reconciliation is limited by poor health and medication literacy. That is that patients are not aware of the medication reconciliation process and do not realise that theirs is an important task in this process. They are not aware of the importance of having a clear and up-to-date insight into their own medication.

The barriers and drivers found in this study are consistent with results of previous similar studies, all carried out in the U.S. [24-27]. These studies also found that: it is crucial that all parties involved have clearly defined roles and responsibilities; that there is a lack of uniformity across hospitals; that pharmacists do not play a significant enough role in the medication reconciliation process; that information was fed back infrequently; and that patients have little knowledge of their medication. The important drivers mentioned include: phased implementation; a multidisciplinary approach where hospital and community caregivers generate a common vision; and collaboration between the involved stakeholders. Several barriers found in these studies, such as ‘medication list not available’, ‘no access to outside records’ and ‘cumbersome hospital systems’ could be overcome with a regional or national electronic patient medication file. Other research also focuses on electronic tools as driving the implementation of medication reconciliation [36]. The importance of patients in medication reconciliation is recognised by Varkey et al., who emphasise the importance of patient education [37].

Strategies can be drawn up to improve the implementation of medication reconciliation based on the barriers and drivers identified. These have been summarised in Table 3. These are found to influence implementation on different levels, for example on patient, professional, and organisational level. Therefore, to improve implementation a multifacitated and multitargeted strategy which intervenes on different levels should be considered. Some of the suggestions mentioned in Table 3 are discussed hereafter in more detail.

**Table 3:** Suggestions for strategies based on barriers and drivers found

**Barriers and drivers**	**Implementation strategy**
A lack of awareness of benefits of bundle	Process mapping of the medication reconciliation process to get insight into inefficiencies
The bundle does not meet the wishes or needs of professionals	Tailoring bundle to local barriers and needs of professionals
Compatibility	Use uniform and electronic forms between departments and between inpatient and outpatient setting
Insufficient knowledge of professionals	Inform, thoroughly, professionals about the medication reconciliation process
	Use a training and implementation toolbox, including tools for
	transferring knowledge and forms for generating feedback
	Generate feedback about professionals’ performance with quality indicators
Feedback	Use a central database for medication errors occurring in inpatient
	and outpatient settings to generate feedback
Collaboration between hospital and community caregivers	Adopt a multidisciplinary team approach including hospital and community caregivers generating a common purpose
	
Limited knowledge of patient	Encourage patient empowerment through medication education
Competitive spirit	Facilitate competition by publishing and comparing the performance of departments
Extra resources to measure indicators	Integrate the measurement of indicators with existing ICT tools
Unavailable information from community pharmacies	Adopt a regional or national electronic medication patient file
during out of office hours	
Task reallocation	Reallocate tasks to those professionals who are best educated to perform medication reconciliation
	Incorporate community pharmacists into the medication reconciliation process, due to their knowledge of comorbidities and medication history
Multiple interventions at once	Synthesise the implementation of different interventions when possible

Professionals with more awareness of the importance of medication reconciliation are more likely to change their performance [38]. An analysis of the process of medication reconciliation gives insight into the current process of care and its inefficiencies. Collecting feedback about the implementation, and about the reduction in medication errors keeps professionals informed and engaged. A lack of clarity about tasks and responsibilities can be resolved with a clear written policy. Research into the effectiveness of task reallocation of the medication history taking to pharmacy technicians should be emphasised. They are most specialised in relation to their lower salary, probably leading to higher cost-effectiveness.

A lack of collaboration between the many health care professionals involved in medication reconciliation can be addressed by a partnership between hospital and community pharmacy providers. This is important to ensure uninterrupted communication both in the inpatient and outpatient settings [24]. Community pharmacies should be considered as a partner in medication reconciliation, especially with regard to high risk patients. Community pharmacies have frequent and direct contact with patients, resulting in a complete overview of patients’ medication history and offers opportunities to educate patients.

Finally, in every aspect of care patient empowerment will become more and more important. Therefore it is essential to create awareness among patient of the importance of carrying an accurate and up-to-date list of medications. Patients should be encouraged to take their own responsibility. They want to be in control of their own care, and thus in control of their medication [24].

A methodological strength of this study is that we applied qualitative methods to explore, in-depth, all possible barriers and drivers. Interviews have proven to be a useful method of providing in-depth information on barriers and drivers with regard to implementation while at the same time exploring and understanding the motivations underlying behaviour [39,40]. The interviews enabled us to identify the most relevant barriers and drivers perceived by the persons who were involved in undertaking the implementation of medication reconciliation. Our analysis of barriers provided detailed information for professionals or organisations, regionally or nationally, to develop multifaceted implementation strategies for improving the implementation process of medication reconciliation.

Even so, several limitations should be considered when interpreting our findings. The selection of interviewees from one hospital and the selection of a limited number of a different kind of health care professionals might raise questions about the generalisability of our findings. The results are, however, consistent with previous studies on this subject. A study including a larger sample in different types of hospitals could be performed to confirm our findings. Secondly, neither patients nor GP’s were involved in this study, while medication reconciliation is a multi disciplinary process. Including the patients’ and GP’s perspective would have strengthened the findings of this study. Finally, the effectiveness, the cost-effectiveness and feasibility of the strategies suggested are unknown and have yet to be tested in well-designed controlled evaluations.

## Conclusions

In conclusion, we identified a wide range of barriers and drivers perceived by health care professionals regarding the implementation of medication reconciliation. This reflects the complexity of implementation, which could be improved if these barriers are adequately addressed in implementation strategies. The feasibility and effectiveness of these strategies should be tested in controlled trails.

## Competing interests

The authors declare that they have no competing interests.

## Authors’ contributions

MZ, SN and HW contributed to the study design and coordination. NS and MZ contributed to the collection and interpretation of the data and drafted the manuscript. NS analysed the data. SN and HW revised the manuscript critically for important intellectual content. All authors read and approved the final manuscript.

## Pre-publication history

The pre-publication history for this paper can be accessed here:

http://www.biomedcentral.com/1472-6963/12/170/prepub
